# Supplementary material for: P2X4 receptor controls microglia activation and favors remyelination in autoimmune encephalitis
Source: EMBO Mol Med. 2018 Jul 4;10(8):e8743. doi: 10.15252/emmm.201708743 (PMC6079537; doi:10.15252/emmm.201708743)
Supplement: Supplementary file 1 — Appendix [file EMMM-10-e8743-s001.pdf]

Appendix: P2X4 receptor controls microglia activation and favours remyelination in autoimmune encephalitis

Table of contents:

Appendix supplementary methods

Appendix Figure S1

Appendix Figure S2

Appendix Figure S3

Appendix Supplementary Methods

T cell proliferation and cytokine production

To measure proliferation, splenocytes were labeled with eFluor670 proliferation dye (5 µM, eBioscience), washed and stimulated with anti-CD3/CD28 (1 µg/ml). On day 3 cells were stained with anti-CD4 (#100509, 1:100, Biolegend) and anti-CD8 (#100732, 1:100, Biolegend). Proliferation was measured by dilution of eFluor670 and quantified as division index using FlowJo Software (Treestar).

To measure cytokine production, splenocytes were stimulated for 4h with phorbol-12-myristat-13-acetat (PMA, 50 ng/ml, Sigma) and ionomycin (1 µM, Sigma). Cells were washed and stained with anti-CD4 (#100509, 1:100, Biolegend), anti-CD8 (#100732, 1:100, Biolegend), anti-γδTCR (#118119, 1:100, Biolegend). Cells were fixed with 4 % paraformaldehyde (PFA, Sigma), permeabilized with 0.5% saponin buffer and stained for anti-IFNγ (#54413, 1:100, BD Bioscience) and anti-IL-17A (#12-7177-81, 1:100, eBioscience) and intracellular cytokines were measured by FACS (LSRFortessa, BD).

Immunochemistry

Primary antibodies were used as follow to: iNOS (#610329, 1:500, BD Bioscience), MRC1 (#ab64693, 1:1000, Abcam), NG2 (#AB5320, 1:500, Abcam), Iba1 (#019-19741, 1:1000; Wako), MBP (#SMI-99P, 1:500, Covance), Olig2 (#MABN50, 1:400, Millipore), P2X7R (#APR-004, 1:100, Alomone), P2X4R (#APR-002, 1:400, Alomone) and extracellular P2X4R (#APR-024, 1:400, Alomone). We used the following secondary antibodies goat anti-rabbit Alexa Fluor 488 (A11008, 1:200, Invitrogen) and goat anti-mouse Alexa Fluor 594 (A11032, 1:200, Invitrogen). For isolectin B4 (IB4) staining, live cultures were incubated for 30 min with IB4 (#FL 1201, 1:100, Vector),

washed with PBS, fixed in 4% PFA in PBS for 20 min and processed for conventional immunocytochemistry.

FACS

CD4-Bv605 (#100547, 1:100, Biolegend), CD8-perCP (#100732, 1:100, Biolegend), γδTCR-Bv650 (#118129, 1:100, Biolegend), CD3e-Bv421 (#100228, 1:100, Biolegend), CD45APC-eFluor780 (#47-0451-82, 1:100, eBioscience), Ly6G-AF700 (#127622, 1:100, Biolegend), CD11b-FITC (#101205, 1:100, Biolegend), CD11c-APC (#17-0114-82, 1:100, eBioscience).

qPCR primers

Sequences for mouse primers used for qPCR

Target gene	**Forward (5'->3')**	**Reverse (5'->3')**
Arg1	GGATTGGCAAGGTGATGGAA	CGACATCAAAGCTCAGGTGAA
Bdnf	TCCAAAGGCCAACTGAAGCA	CTGCAGCCTTCCTTGGTGTA
Ccl2	AGCAGCAGGTGTCCCAAA	TTCTTGGGGTCAGCACAGAC
Ccr7	GTGGTGGCTCTCCTTGTCA	GGTATTCTCGCCGATGTAGTCA
Chi3l3	GCCCACCAGGAAAGTACACA	CCTCAGTGGCTCCTTCATTCA
Clec7a	ACCACAAGCCCACAGAATCA	AGGAAGGCAAGGCTGAGAAA
Foxp3	ACCACACTTCATGCATCAGCTC	GGCTGGGTTGTCCAGTGGAC
Ifnγ	TAACTATTTTAACTCAAGTGGCATAGATGTG	GCCAGTTCCTCCAGATATCCAAG
Il10	AAAGGACCAGCTGGACAACA	TAAGGCTTGGCAACCCAAGTA
Il12a	AAACCAGCACATTGAAGACC	GGAAGAAGTCTCTCTAGTAGCC
Il1b	TGGCAACTGTTCCTGAACTCA	GGGTCCGTCAACTTCAAAGAAC
Il4	ACGGAGATGGATGTGCCAAA	GAAGCACCTTGGAAGCCCTA
Il6	CGATGATGCACTTGCAGAAA	ACTCCAGAAGACCAGAGGAA
Irf5	TGATGTCAAACCCCGAGAGAA	GAACATCTCCAGCAGCAACC
Irf8	GATATGCCGCCTATGACACA	CCCGTAGTAGAAGCTGATGAC
Jak3	CATAGAGGACGTGGACACTCAA	TGACATGTCTCCAGCCCAAA
Marco	TTCTGTCGCATGCTCGGTTA	TTGTCCAGCCAGATGTTCCC
Mbp	CCCTCACAGCGATCCAAGTA	CTCTGTGCCTTGGGAGGAA
Mr1	GCTCGCTGTATTCTTGGTGAA	ACCAGGATCGGAAACAGCTA
Mrc1	CACAAAGCCATGCTGTAGTACC	GTAAAACCCATGCCGTTTCCA
Msx3	CTCCAGTCGCGCACTCTT	CCGTGGTTTGCGATTGGTTT
Nos2	GAGGAGCAGGTGGAAGACTA	GGAAAAGACTGCACCGAAGATA

P2rx4	TTTGCGATTCAGACGCCAAC	ATGGAACACACCTTCCAGTCC
Ptgs2	CTTCTCCCTGAAGCCGTACA	TGTCACTGTAGAGGGCTTTCAA
Retnla	ATCCCTCCACTGTAACGAAGAC	ACAAGCACACCCAGTAGCA
Ror	ACTGAAAGCAGGAGCAATGGAAG	TTCAAAAAAGACTGTGTGGTTGTTG
Stat1	GCAGGTGTTGTCAGATCGAAC	ATGCACGGCTGTCGTTCTA
Stat3	TGGGCATCAATCCTGTGGTA	CCAATTGGCGGCTTAGTGAA
Stat6	TGACTTTCCACAACGCCTAC	CATCTGAACCGACCAGGAAC
Tgfb1	GCTGCGCTTGCAGAGATTAA	GTAACGCCAGGAATTGTTGCTA
Tnf	GGGTGATCGGTCCCCAAA	TGAGGGTCTGGGCCATAGAA

Housekeeping gene	**Forward (5'->3')**	**Reverse (5'->3')**
B2m	ACTGACCGGCCTGTATGCTA	ATGTTCGGCTTCCCATTCTCC
Gapdh	AGACGGCCGCATCTTCTT	TTCACACCGACCTTCACCAT
Hprt	CAGTACAGCCCCAAAATGGTTA	AGTCTGGCCTGTATCCAACA
Ppia	AGGGTTCCTCCTTTCACAGAA	TGCCGCCAGTGCCATTA

Sequences for rat primers used for qPCR

Target gene	**Forward (5'->3')**	**Reverse (5'->3')**
Arg1	GTGAAGAACCCACGGTCTGTG	GAGATGCTTCCAATTGCCATACTG
Ccl2	GTGCTGTCTCAGCCAGATGCA	GCTGCTGGTGATTCTCTTGTAGTT
Mrc1	AAGTTTAAGCACTGGCTGGCA	CAGGTTCTGATGATGGACTTCCTG
Nos2	GAGATTTTTCACGACACCCTTCAC	CATGCATAATTTGGACTTGCAAG

Housekeeping gene	**Forward (5'->3')**	**Reverse (5'->3')**
Cyclophilin A	CAAAGTTCCAAAGACAGCAGAAAA	CCACCCTGGCACATGAATC
Gapdh	GAAGGTCGGTGTCAACGGATTT	CAATGTCCACTTTGTCACAAGAGA
Hprt	ATGGACTGATTATGGACAGGACTGA	ACACAGAGGGCCACAATGTG

Appendix Figures

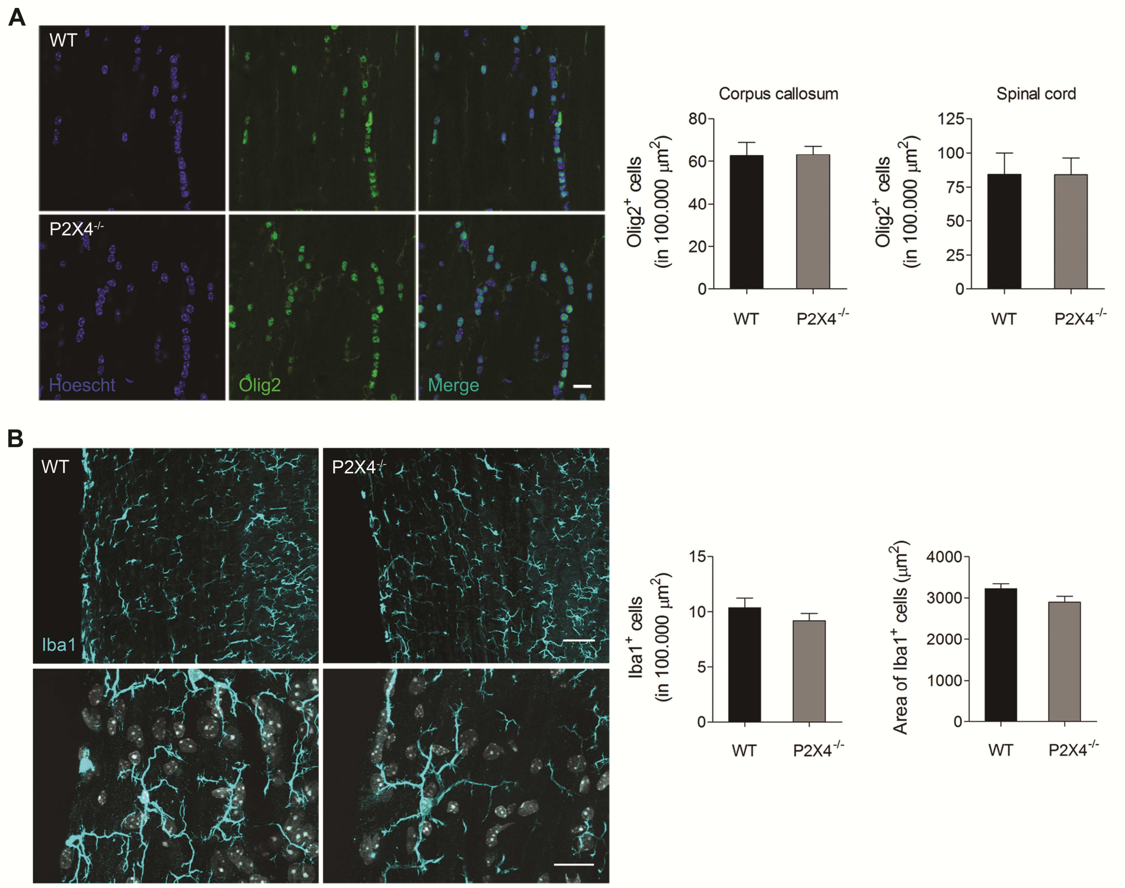

Appendix Figure S1. Microglia and oligodendroglial characterization of WT and $P2X4^{-/-}$ mice.

A Immunohistochemistry analysis of Olig2, a marker of oligodendrocytes in corpus callosum and spinal cord of WT and $P2X4^{-/-}$ mice. Images show representative fields in corpus callosum (n = 3). Scale bar = 20 µm.

B Immunohistochemistry analysis of Iba1, a marker of microglia in the spinal cord of WT and $P2X4^{-/-}$ mice (n = 3). Scale bar = 50 (top) and 20 (bottom) µm.

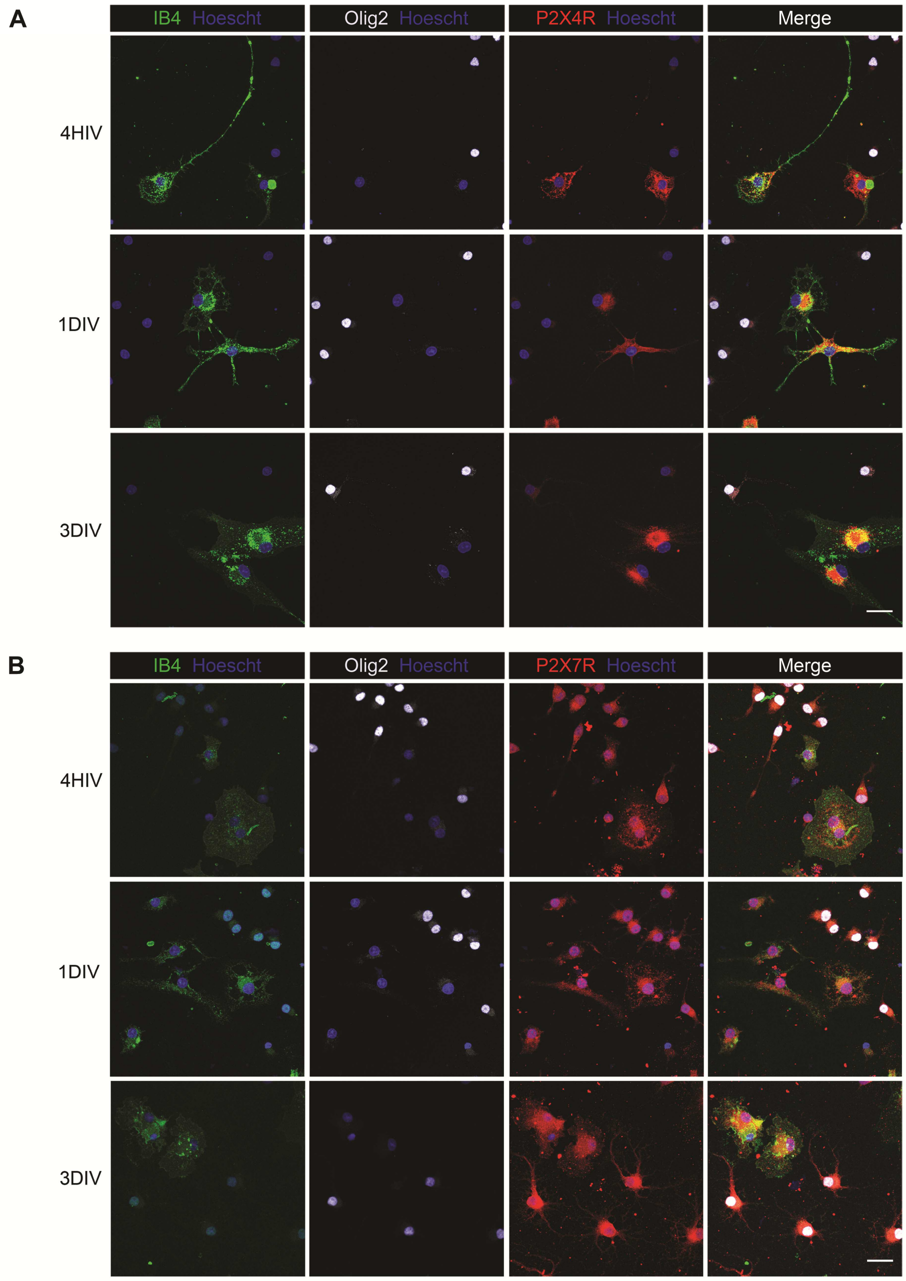

Appendix Figure S2. P2X4R is expressed predominantly in microglia.

A-B) Representative images of microglia-OPCs coculture stained for P2X4R (A; red) or P2X7R (B; red), Olig2 (white), isolectin B4 (IB4; green) and Hoechst (blue) at different stages of oligodendrocyte development, 4 hours in vitro (HIV), 1 day in vitro (DIV) and 3 DIV. Scale bar = 20 µm

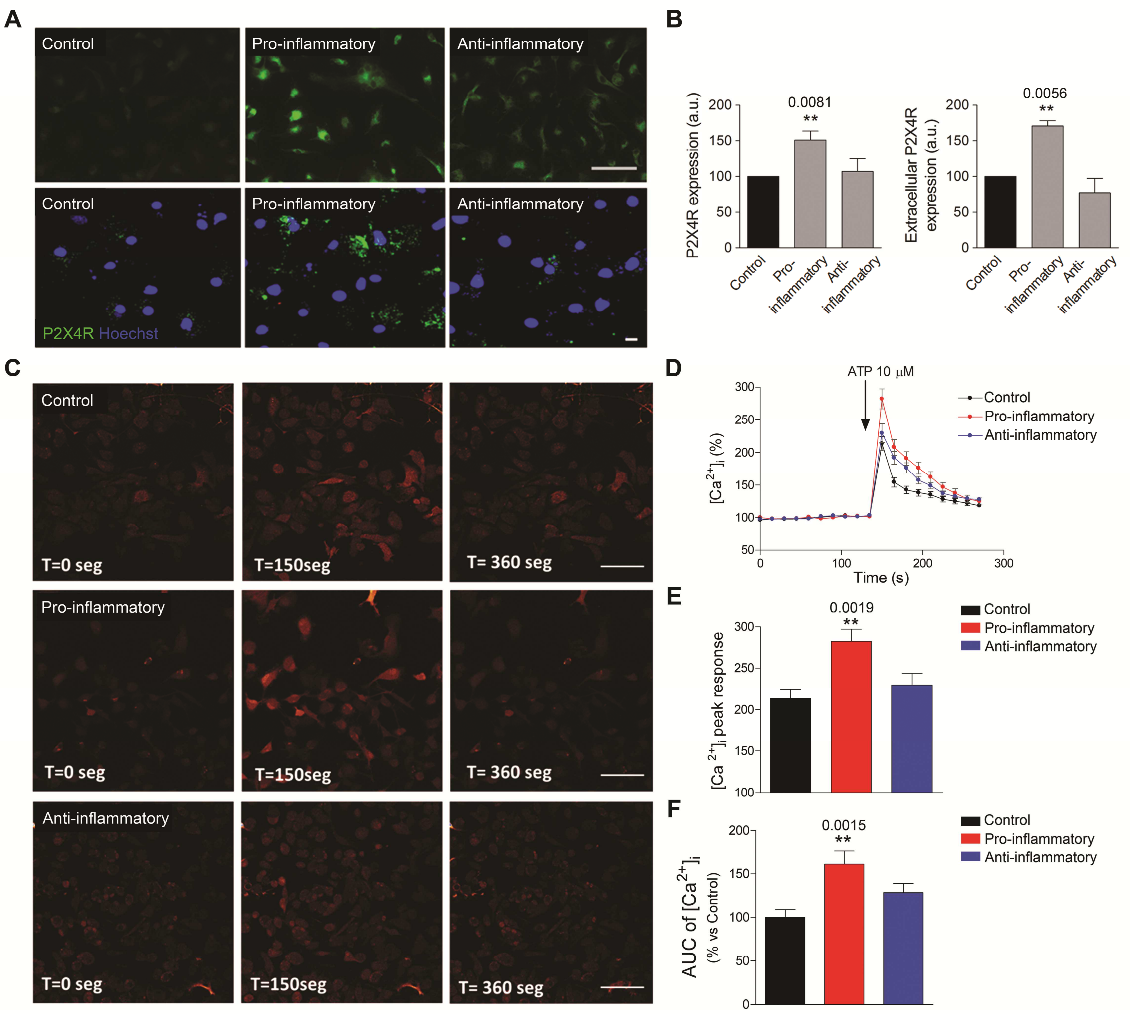

Appendix Figure S3. P2X4R expression and function in differentially polarized microglia.

A, B) Total P2X4R (green, top) (n = 5) or extracellular P2X4R (green, bottom) (n = 3) expression in control, pro-inflammatory and anti-inflammatory microglia phenotypes. Scale Bar= 80 µm (top) and 10 µm (bottom).

C-F) Calcium responses to ATP (10 µM) in control, pro-inflammatory and anti-inflammatory microglia: Representative images at 0, 150 and 360 seconds. ATP was applied at t=150s (n = 150 cells from 3 different cultures). Scale Bar= 80 µm.
