# Supplementary material for: P2X4 receptor controls microglia activation and favors remyelination in autoimmune encephalitis
Source: EMBO Mol Med. 2018 Jul 4;10(8):e8743. doi: 10.15252/emmm.201708743 (PMC6079537; doi:10.15252/emmm.201708743)
Supplement: Supplementary file 2 — Expanded View Figures PDF [file EMMM-10-e8743-s002.pdf]

Expanded View Figures

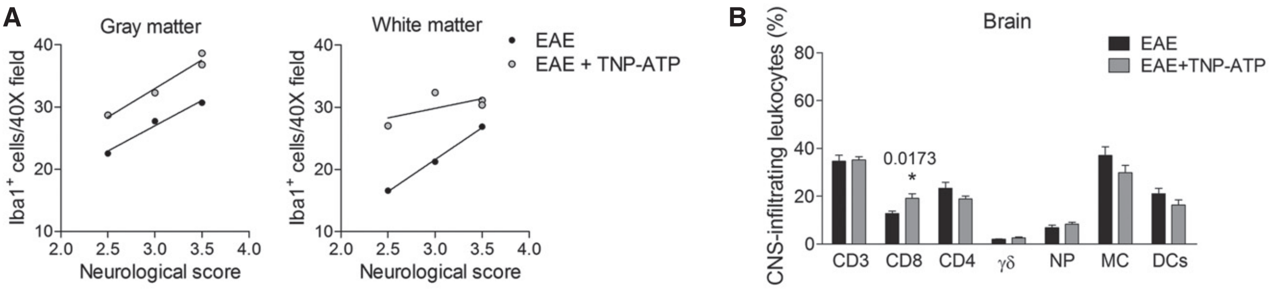

Figure EV1. Immune cell analysis in EAE and TNP-ATP-treated EAE mice.

A Correlation between the neurological score and the number of Iba1$^+$ cells in gray matter and white matter of the spinal cord.

B Flow cytometric quantification of immune cell infiltrates (gating as in Fig 2E) in the brain of TNP-ATP-treated or non-treated mice analyzed at the peak of symptoms after EAE induction (n = 8). Data are presented as mean ± s.e.m. and were analyzed by Student's t-test. *$P < 0.05$.

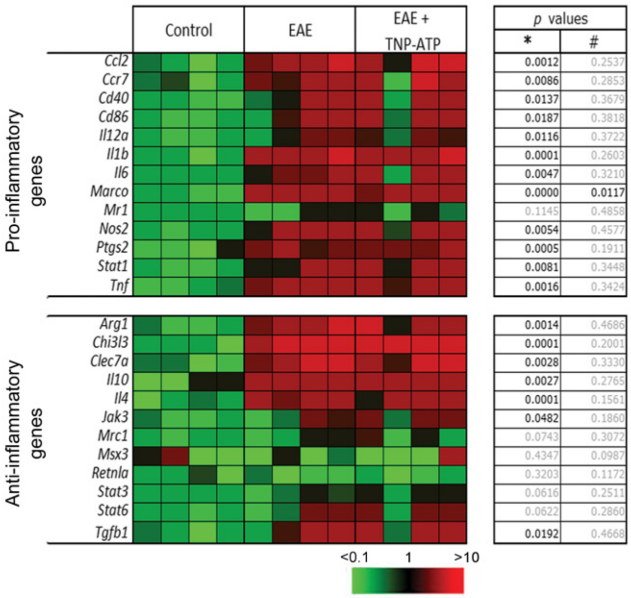

Figure EV2. Heatmap of pro-inflammatory and anti-inflammatory genes at EAE peak.

Expression of most pro-inflammatory genes and some anti-inflammatory genes was increased in the spinal cord of EAE mice (n = 3) at the peak phase, but TNP-ATP did not induce significant changes (n = 3). Tables indicate statistical significance between control and EAE (*) and between EAE and EAE + TNP-ATP (#). Statistics were performed with Student's t-test.

© 2018 The Authors

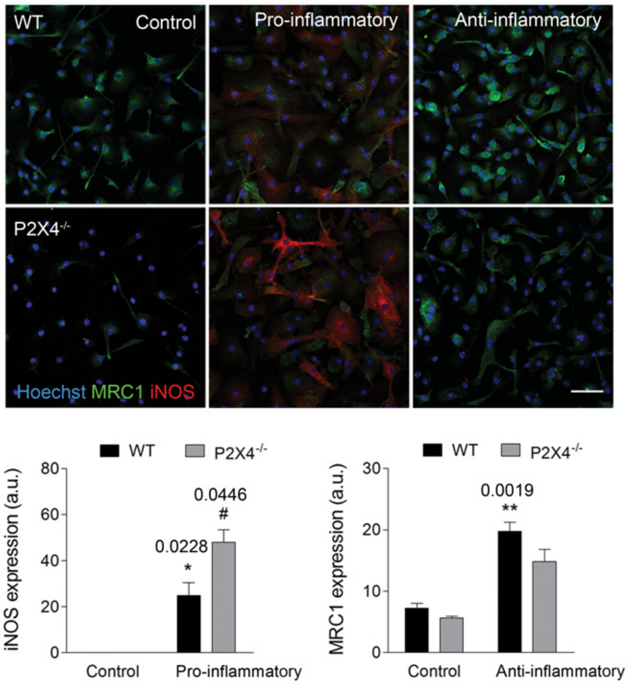

Figure EV3. Pro-inflammatory and anti-inflammatory polarization in wild-type and P2X4$^{-/-}$ microglia.

Staining for iNOS (red) and mannose receptor (MRC1, green) in control, pro-inflammatory, and anti-inflammatory microglia from wild-type and P2X4$^{-/-}$ mice. Scale bar = 50 μm (n = 4 experiments performed in triplicate). Data are presented as mean ± s.e.m. and were analyzed by Student's t-test. $^{*/\#}P < 0.05$, $^{**}P < 0.01$. Symbols indicate significance versus control (*) or versus pro-/anti-inflammatory microglia (#).

 © 2018 The Authors

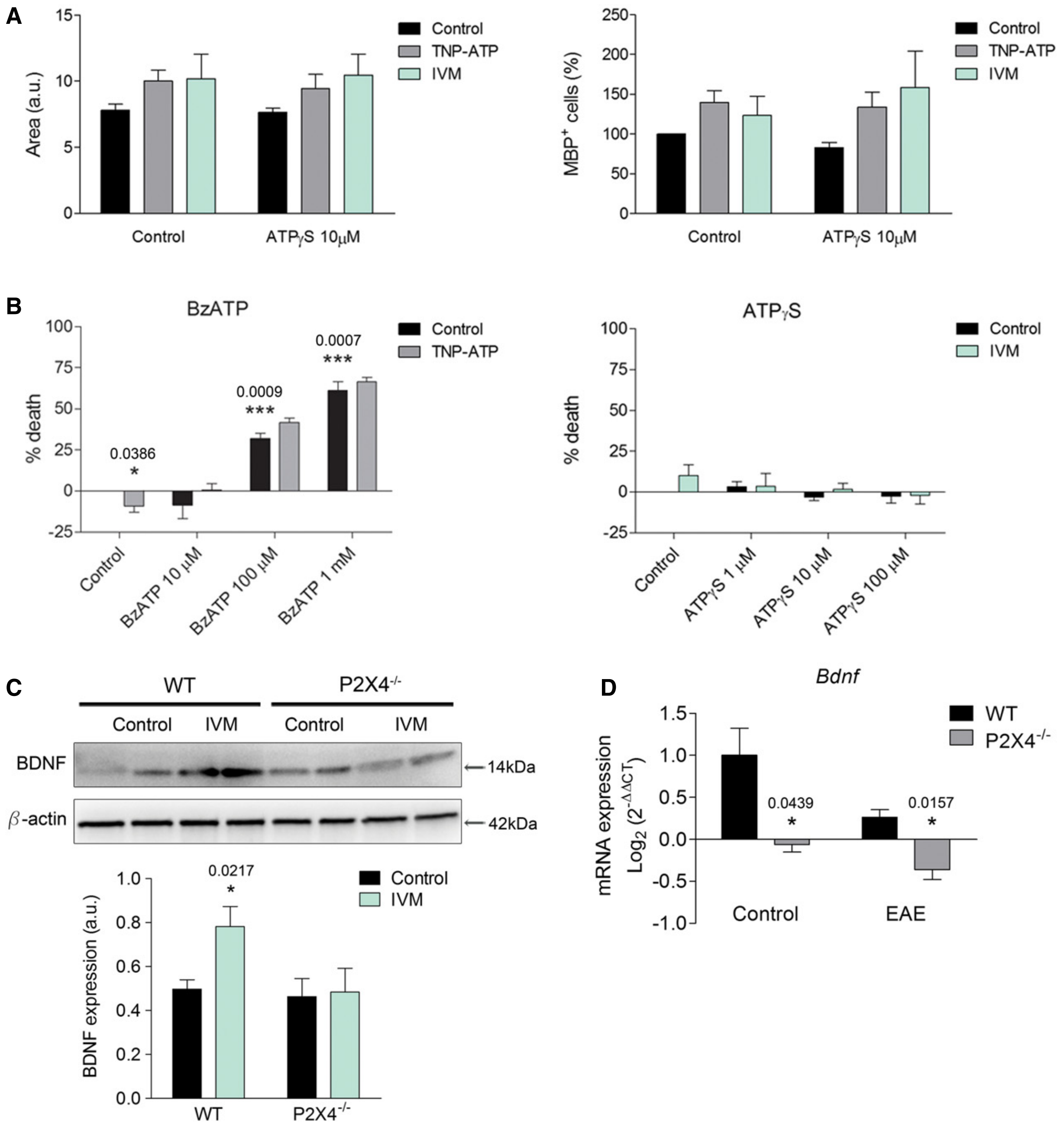

Figure EV4. Functional role of P2X4R in oligodendrocytes in vitro.

A Effect of ATPγS (10 μM) ± ivermectin or TNP-ATP (10 μM) on oligodendrocyte differentiation quantified on the basis of the number of MBP^+ cells/total cell number and the area of the cells. Area was calculated with ImageJ software (n = 3).

B Oligodendrocyte cell viability after 24-h incubation with BzATP, a broad-spectrum agonist of purinergic receptors, in the absence or presence of TNP-ATP (10 μM) (left) or ATPγS (right) in the absence or presence of IVM (3 μM) (n = 3).

C Western blot analysis of BDNF in control and IVM-treated wild-type and $P2X4^{-/-}$ microglia (n = 3).

D *Bdnf* mRNA levels in the spinal cord of WT and $P2X4^{-/-}$ mice in control and after EAE induction (n = 4 mice/group).

Data information: Data are presented as mean ± s.e.m. and were analyzed by one-way ANOVA (B) and Student's t-test (C, D). *$P < 0.05$, ***$P < 0.001$.
Source data are available online for this figure.

© 2018 The Authors

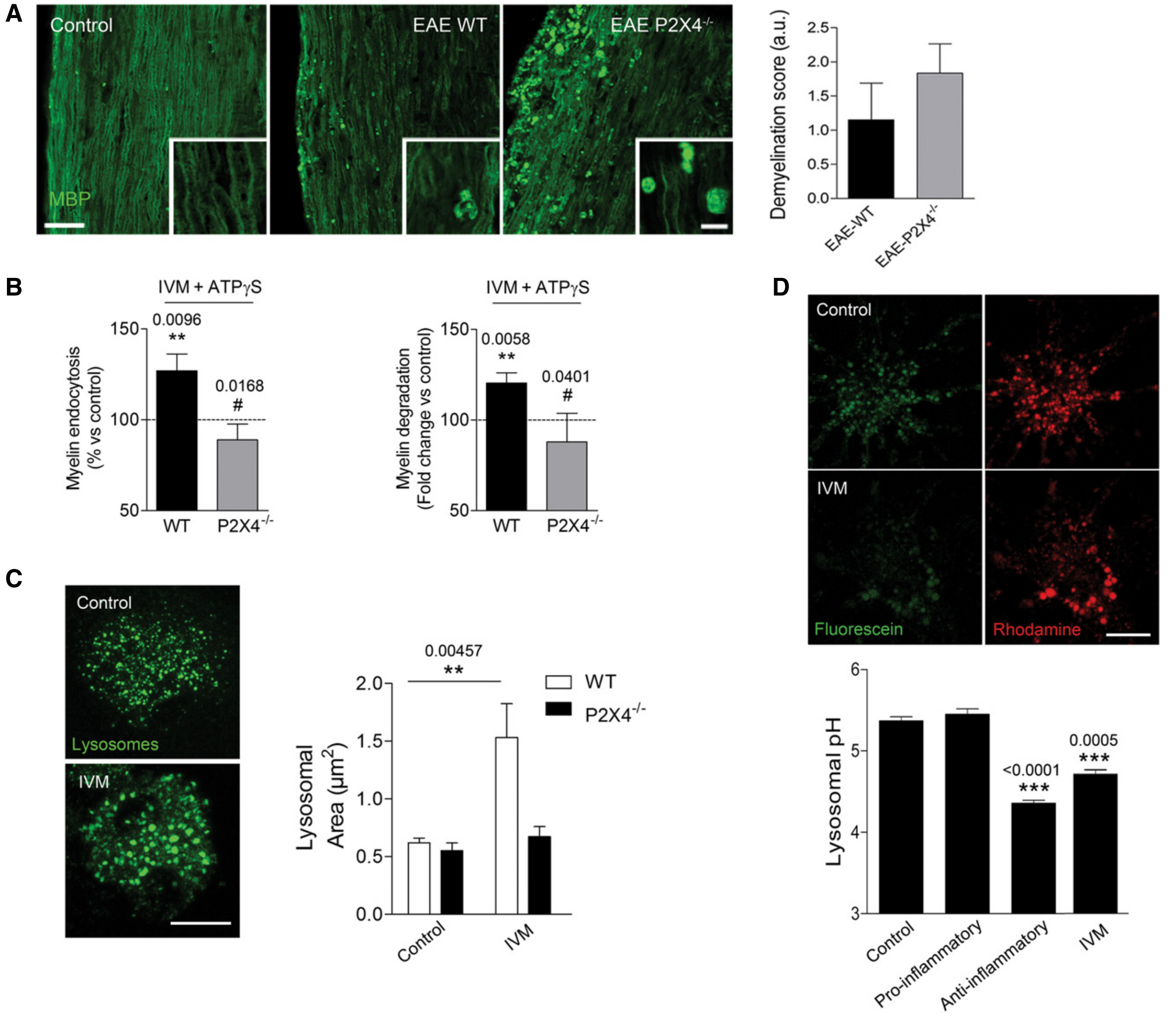

Figure EV5. Role of P2X4R in myelin phagocytosis and lysosome function.

A Spinal cord sections of wild-type (n = 6) and P2X4$^{-/-}$ mice (n = 7) 35 days after EAE induction stained for MBP. Scale bar = 50 and 10 (inset) μm. Inset images come from different fields at higher magnification. Right, demyelination score analyzed in MBP-stained sections.

B Effect of IVM (3 μM) on myelin endocytosis (1 h) and degradation (3 days) in microglia from wild-type and P2X4$^{-/-}$ mice (n = 3).

C Treatment with IVM (3 μM; 16 h) increased late endosome–lysosome (LEL) size in microglia from wild-type and P2X4$^{-/-}$ mice. Representative images and histogram for LEL size distributions under the conditions indicated (n = 20 cells from two independent experiments). Scale bar = 10 μm.

D IVM-treated microglia (3 μM) and anti-inflammatory microglia showed an acidic shift in lysosomal pH, as analyzed on the basis of fluorescein/rhodamine–dextran ratio (n = 20 cells from two independent experiments). Scale bar = 10 μm.

Data information: Data are presented as mean ± s.e.m. and were analyzed by one-way ANOVA. $^{\#}P < 0.05$, $^{**}P < 0.01$, $^{***}P < 0.001$. Symbols indicate significance versus control (*) or versus pro-inflammatory microglia (#).

© 2018 The Authors
