# Supplementary figures and images for: P2X4 receptor controls microglia activation and favors remyelination in autoimmune encephalitis
Source: EMBO Mol Med. 2018 Jul 4;10(8):e8743. doi: 10.15252/emmm.201708743 (PMC6079537; doi:10.15252/emmm.201708743)

# Figure EV4

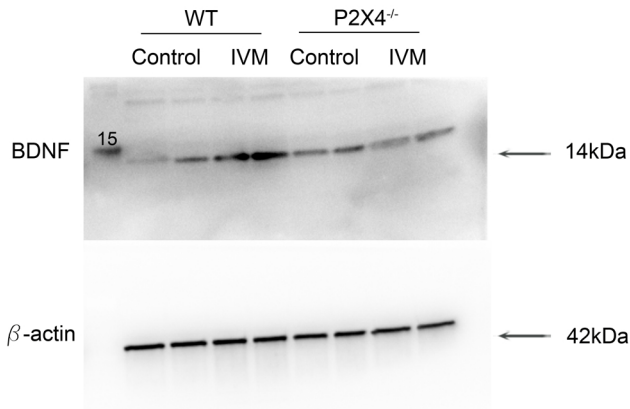

Supplement: Supplementary file 3 — Source Data for Expanded View [file EMMM-10-e8743-s005.zip › emmm201708743-sup-0005-SDataFigEV4.pdf]

Figure 6D

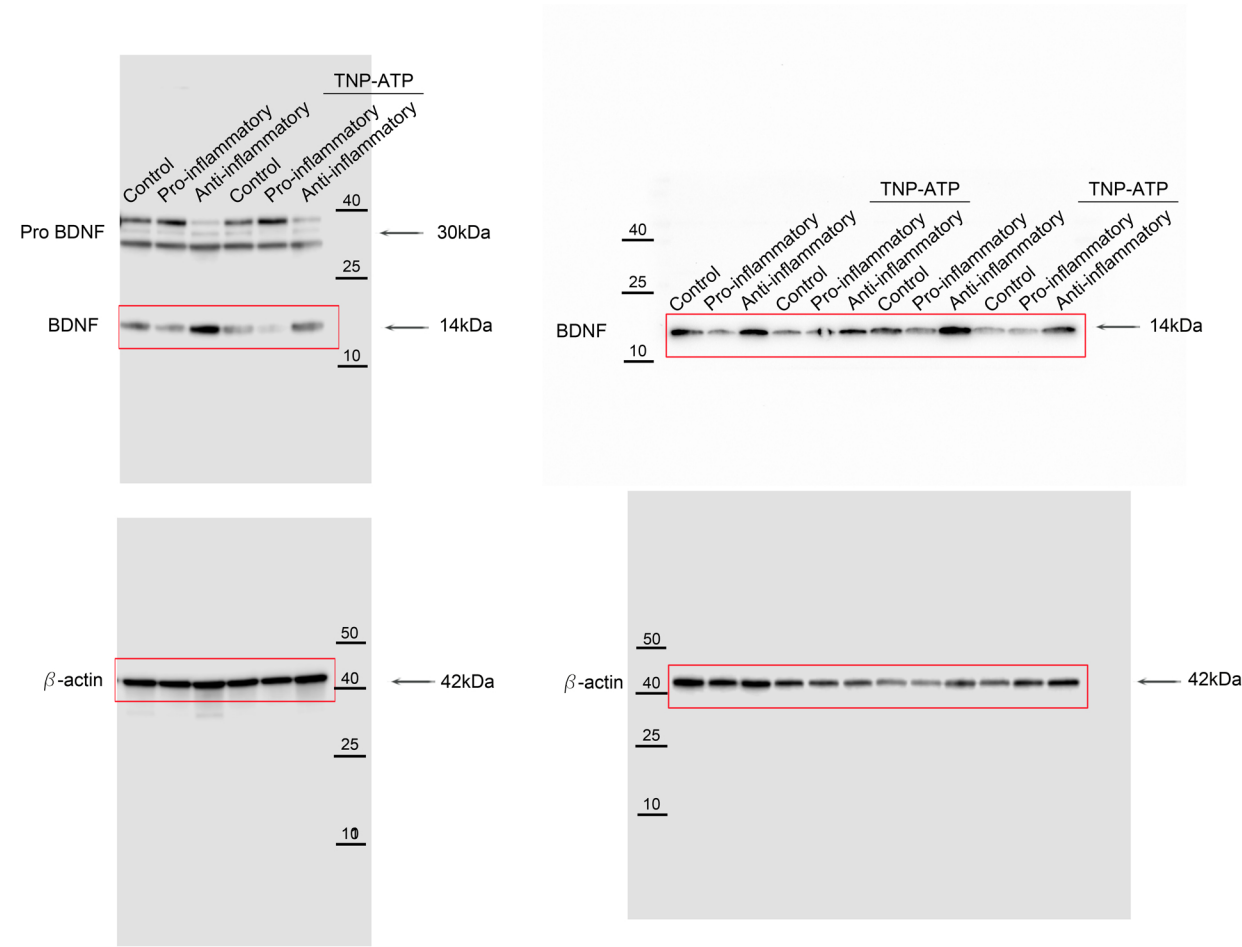

Figura 6F

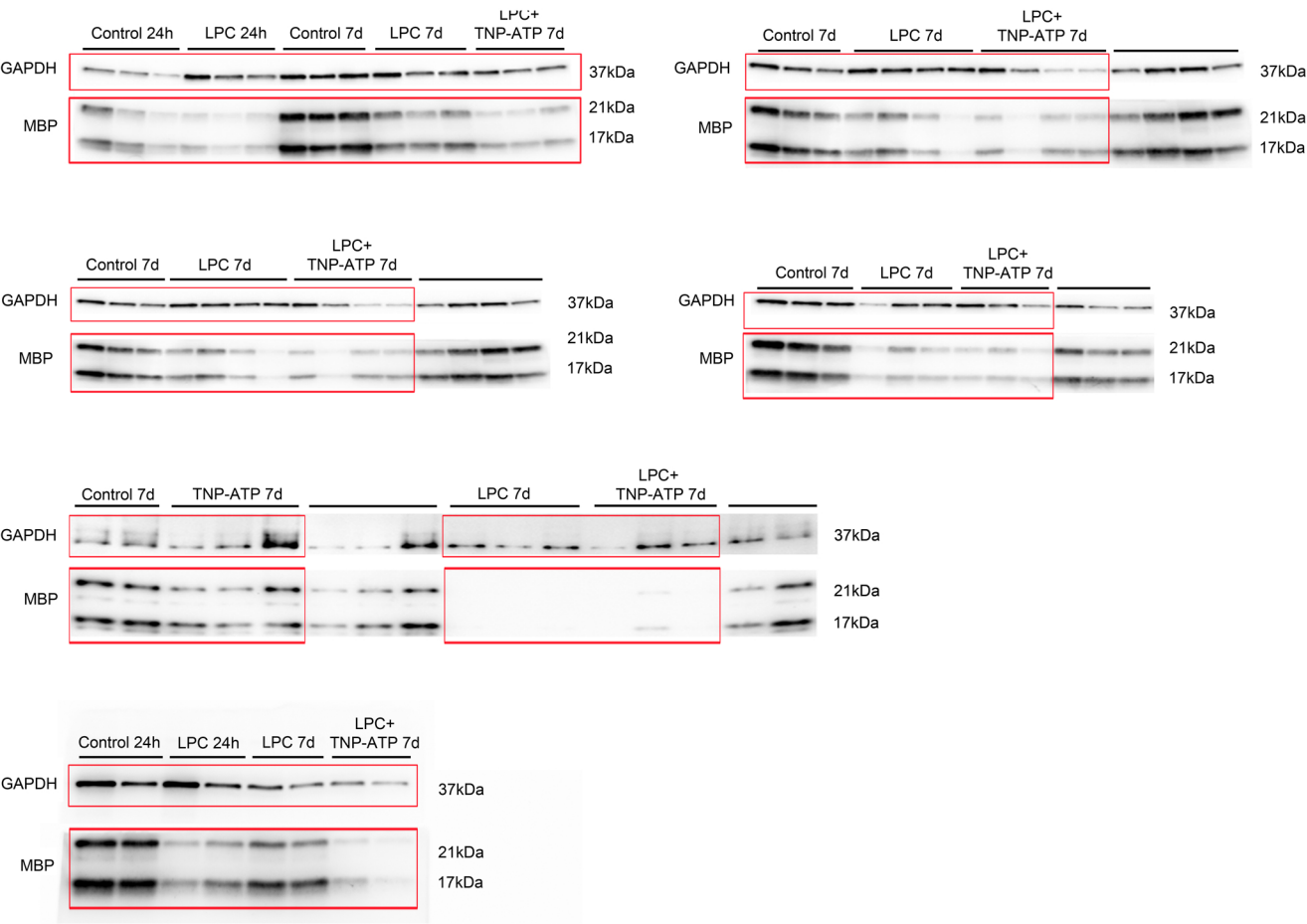

Supplement: Supplementary file 5 — Source Data for Figure 6 [file EMMM-10-e8743-s003.pdf]

Figure 8G

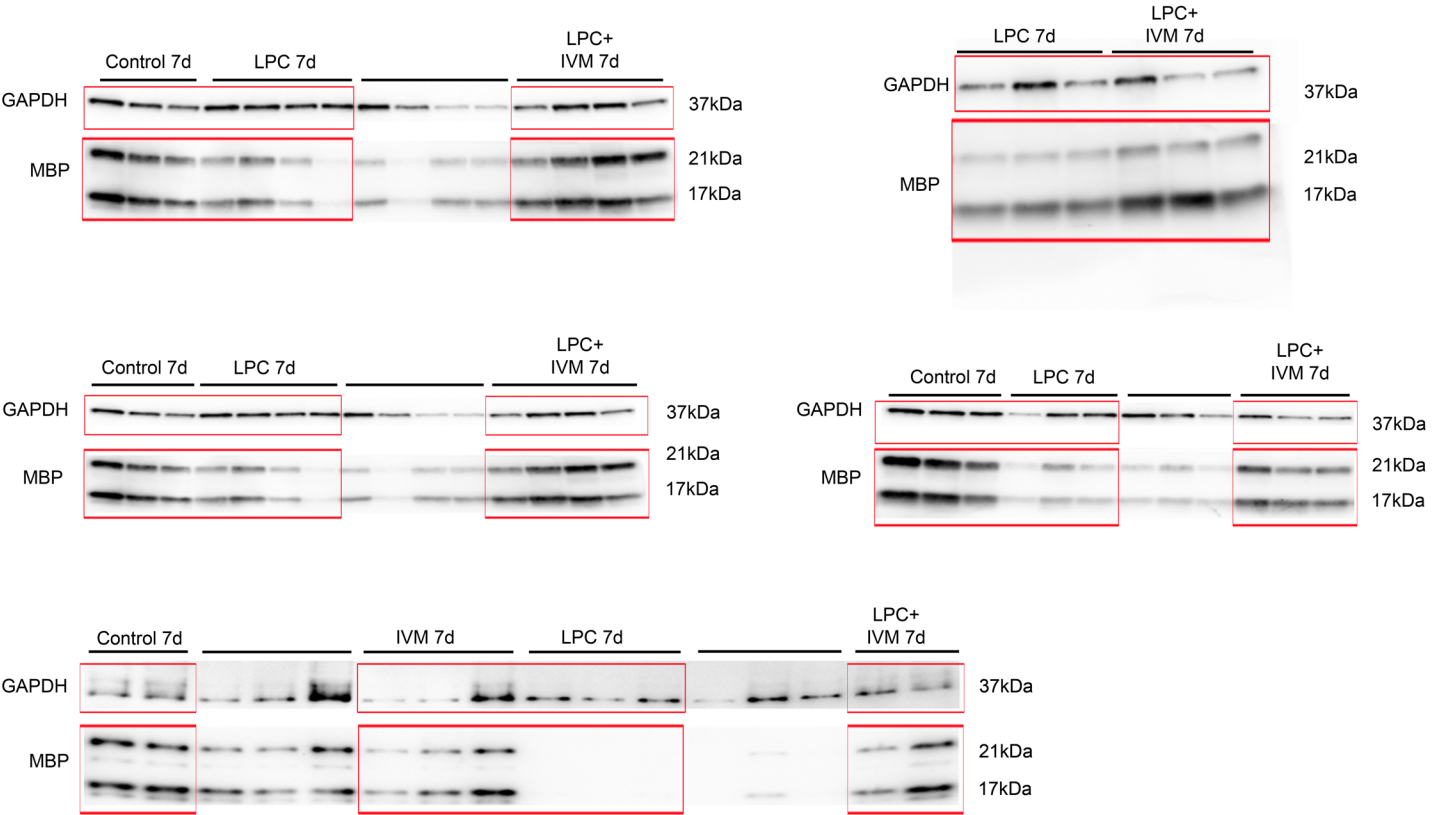

Supplement: Supplementary file 6 — Source Data for Figure 8 [file EMMM-10-e8743-s004.pdf]
